# An evaluation of the Diamat HPLC analyser for simultaneous determination of haemoglobins A_2_ and F

**DOI:** 10.1155/S1463924689000520

**Published:** 1989

**Authors:** Andrea Mosca, Assunta Carpinelli, Rita Majavacca, Angelo Cantu'-Rajnoldi, Massimo Garatti, Renata Paleari, Maurizio Ferrari, Vittorio Agape, Liliana Maccioni, Sandra Pisano, Renzo Galanello

**Affiliations:** 1 Universita' degli Studi Dip. Scienze e Tecnologie Biomediche Via Olgettina 60 Milano 20132 Italy; 2 I.C.P. Laboratorio di Ricerche Cliniche Milano Italy; 3 Ist. Sc. H. S. Raffaele Lab. Centrale Milano Italy; 4 Universitá degli Studi Ist. Clin. Biologia Eta' Evolutiva Cagliari Italy; 5 Dip. Scienze Tecnologie Biomediche Via Olgettina 60 Milano 20132 Italy

## Abstract

The authors describe a modification of the instrumental parameters of the Diamat fully automated HPLC system for Hb A_2_ assay (Bio-Rad Laboratories, Milan, Italy) in order to obtain simultaneous determination of Hb A_2_ and Hb F.

Hb A_2_ and Hb F measurements are reproducible (within-run CV 2.6%, with Hb A_2_2.7%; 5.1%, with Hb F 1.3%) and accurate (from a comparison with two microchromatographic techniques for Hb A_2_: r = 0.9639 and 0.9755; with two alkali denaturation procedures for Hb F: r = 0.9990 and 0.9952; with radial immunodiffusion, r = 0.9877). Assay linearity has been confirmed for Hb A_2_ concentrations between 0 and 6.0%, and for Hb F between 0 and 60%. The data obtained from the analysis of some pathological samples for Hb Bart's, Hb H, Hb J Sardegna, Hb Lepore and Hb S are in agreement with cellulose acetate electrophoresis analysis.

The Hb A_2_ reference intervals for normals (N = 597) and Beta-thalassemia carriers (N = 200) are respectively (95% limits) 2.02-3.27 and 3.92-5.90 in % units. Hb F values measured in normals (N = 968), in β-thal carriers (N = 302) and in δβ-thal carriers (N  =3) have been found to be consistent with the usual diagnostic parameters.

Some minor limitations emerged: the most relevant concerns Hb A_1c_, which is overestimated with respect to a reference method (y = 1.217x + 0.16; N = 79; r = 0.9235). A probable interference from labile fractions is responsible for this Hb A_1c_ inaccuracy.


Journal of Automatic Chemistry Vol. 11, No. 6 (November–December 1989), p. 273–279

### An evaluation of the Diamat HPLC analyser for simultaneous determination of haemoglobins $A_2$ and F

**Andrea Mosca,† Assunta Carpinelli**
*Universita' degli Studi, Dip. Scienze e Tecnologie Biomediche, Via Olgettina 60, 20132 Milano, Italy*

**Rita Majavacca, Angelo Cantu'-Rajnoldi, Massimo Garatti**

*I.C.P., Laboratorio di Ricerche Cliniche, Milano, Italy*

**Renata Paleari, Maurizio Ferrari, Vittorio Agape**
*Ist. Sc. H. S. Raffaele, Lab. Centrale, Milano, Italy*

**Liliana Maccioni, Sandra Pisano and Renzo Galanello**
*Universitá degli Studi, Ist. Clin. Biologia Eta' Evolutiva, Cagliari, Italy*

*The authors describe a modification of the instrumental parameters of the Diamat fully automated HPLC system for Hb $A_2$ assay (Bio-Rad Laboratories, Milan, Italy) in order to obtain simultaneous determination of Hb $A_2$ and Hb F.*

*Hb $A_2$ and Hb F measurements are reproducible (within-run CV 2·6%, with Hb $A_2$ 2·7%; 5·1%, with Hb F 1·3%) and accurate (from a comparison with two microchromatographic techniques for Hb $A_2$: r = 0·9639 and 0·9755; with two alkali denaturation procedures for Hb F: r = 0·9990 and 0·9952; with radial immunodiffusion, r = 0·9877). Assay linearity has been confirmed for Hb $A_2$ concentrations between 0 and 6·0%, and for Hb F between 0 and 60%. The data obtained from the analysis of some pathological samples for Hb Bart's, Hb H, Hb J Sardegna, Hb Lepore and Hb S are in agreement with cellulose acetate electrophoresis analysis.*

*The Hb $A_2$ reference intervals for normals (N = 597) and Beta-thalassemia carriers (N = 200) are respectively (95% limits) 2·02–3.27 and 3·92–5·90 in % units. Hb F values measured in normals (N = 968), in β-thal carriers (N = 302) and in δβ-thal carriers (N = 3) have been found to be consistent with the usual diagnostic parameters.*

*Some minor limitations emerged: the most relevant concerns Hb $A_{1c}$, which is overestimated with respect to a reference method (y = 1·217x + 0·16; N = 79; r = 0·9235). A probable interference from labile fractions is responsible for this Hb $A_{1c}$ inaccuracy.*

### Introduction

The determination of the minor haemoglobins, Hb $A_2$ and Hb F, in whole blood are of considerable diagnostic use for the characterization of several thalassaemic syndromes and for the correct diagnosis of several haemoglobinopathies [1]. It is also known that elevated levels of Hb F can be associated with neoplastic conditions, anaemias and leukaemias of different etiologies [2]. Therefore, a variety of laboratory techniques have been developed to accurately measure these minor haemoglobins. The most commonly used are ion-exchange chromatography [3], on microcolumns or with HPLC [4–7], for Hb $A_2$, and radial immunodiffusion [8] and alkali denaturation [9–11] for Hb F.

This report presents the authors' experience with a new HPLC method performing a simultaneous determination of Hb $A_2$ and Hb F. This is obtained by using a fully automated HPLC analyser (Diamat, Bio-Rad Laboratories, Segrate, Milan, Italy), originally dedicated to the analysis of glycated haemoglobins [12–14], and later adapted to Hb $A_2$ determination [15].

By introducing a slight modification to the buffer elution times recommended by the manufacturer for Hb $A_2$ determination, the method was adapted for measuring Hb $A_2$ and Hb F simultaneously.

The data reported here confirm the utility of the technique to haematological practice. Some minor limitations are also discussed.

### Materials and methods

### *Samples*

Whole blood samples (anticoagulated with potassium EDTA, 1 g/l) were selected from routine laboratory samples for most of the analytical tests. Informed consent was obtained from those subjects studied for the evaluation of reference values.

Stabilized Hb $A_2$ and Hb F liquid control materials were obtained by adding ethylene glycol (0·35 volume fraction) to haemolysates prepared by a standard tetrachloromethane lysis procedure [16]. These solutions total Hb 100 ± 20 g/l) were stored at −20 °C and used for long-term precision estimates. This was carried out by assaying two haemolysates, with low and high Hb $A_2$ and Hb F concentrations, over periods of about one month (the Hb $A_2$ test) and three months (the Hb F test).

### Procedures

### *Diamat HPLC analysis*

This method basically consists of a cation-exchange liquid chromatography developed with three tris-phosphate buffers of increasing ionic strength at a

† Correspondence to Dr Andrea Mosca, Dip. Scienze Tecnologie Biomediche, Via Olgettina 60, 20132 Milano, Italy.

0142–0453/89 $3.00 © Taylor & Francis Ltd.

controlled temperature of 23 °C. The eluate is read at 415 and 690 nm. For the analysis five μl of whole blood are diluted, by means of the dilutor supplied with the apparatus, with 1 ml of haemolizing reagent. The haemolysate is centrifuged at 10 000 *g* for 1 min and is then loaded into the sample compartment, which is kept at between 2 and 8 °C. Twenty μl are injected into the column for each run.

Figure 1 shows the modifications made to the instrumental parameters recommended by the manufacturer in order to obtain a resolution of Hb F from Hb $A_{1c}$. The best Hb F isolation is achieved by prolonging the buffer 1 elution to 2 min (figure 1[*c*]); there is no effect if the elution of buffer 2 is prolonged from 6·4 to 9·4 min. Therefore, after these tests, it was decided to adopt the following instrumental parameters: injection interval = 170; injection volume = 2; stepwise time 1 = 20; stepwise time 2 = 77; stepwise time 3 = 140; stop time = 9900; off time = 800; response time = 1·00 (for all the minor fractions).

The flow is critical for optimal resolution of the Hb F peak: a flow of 1 ml/min is obtained by setting the internal knob to a position between 25 and 30. The operating pressure, under such conditions, is between 55 and 60 kg/cm$^2$. By using these parameters the haemaglobins are eluted in the following order (retention times in parentheses, and given in min): Hb $A_{1a}$ and $A_{1b}$ (1·7), $A_{1c}$ (3·1), F (3·9), $A_0$ (7·5), $A_2$ (9·9); each run takes approximately 16·5 min.

Columns and reagents used within this evaluation were from Bio-Rad HF and KK batches. All the chromatograms in figures 1–3 have been redrawn from the original paper print-out.

*Reference methods*

Alternative chromatographic procedures for Hb $A_2$ have been performed either according to ICSH recommendations [3, 17] or by using disposable microcolumns (Beta-Thal Quick Column, Helena Laboratories, Milan, Italy). The average within-run imprecision for Hb $A_2$ determinations by these methods (data from two different laboratories) are:

(1) ICSH method: CV 14·3% (Hb $A_2$ 2·58%) and 3·7% (Hb $A_2$ 5·86%).

(2) Microcolumns: CV 2·8% (Hb $A_2$ 2·49%) and 1·2% (Hb $A_2$ 5·22%).

As Hb F reference methods the following were used:

(*a*) Alkali denaturation, method 1 [9].

(*b*) Alkali denaturation, method 2 [11].

(*c*) Radial immunodiffusion, for samples with Hb F lower than 12%.

(*d*) Diamat analysis for Hb $A_{1c}$ (see later).

The within-run imprecisions of these methods (as CV) for Hb F concentrations between 0·8 and 2·2% are: 7·3% (method *a*), 8·0% (method *b*), 4·3% (method *c*) and 3·3% (method *d*).

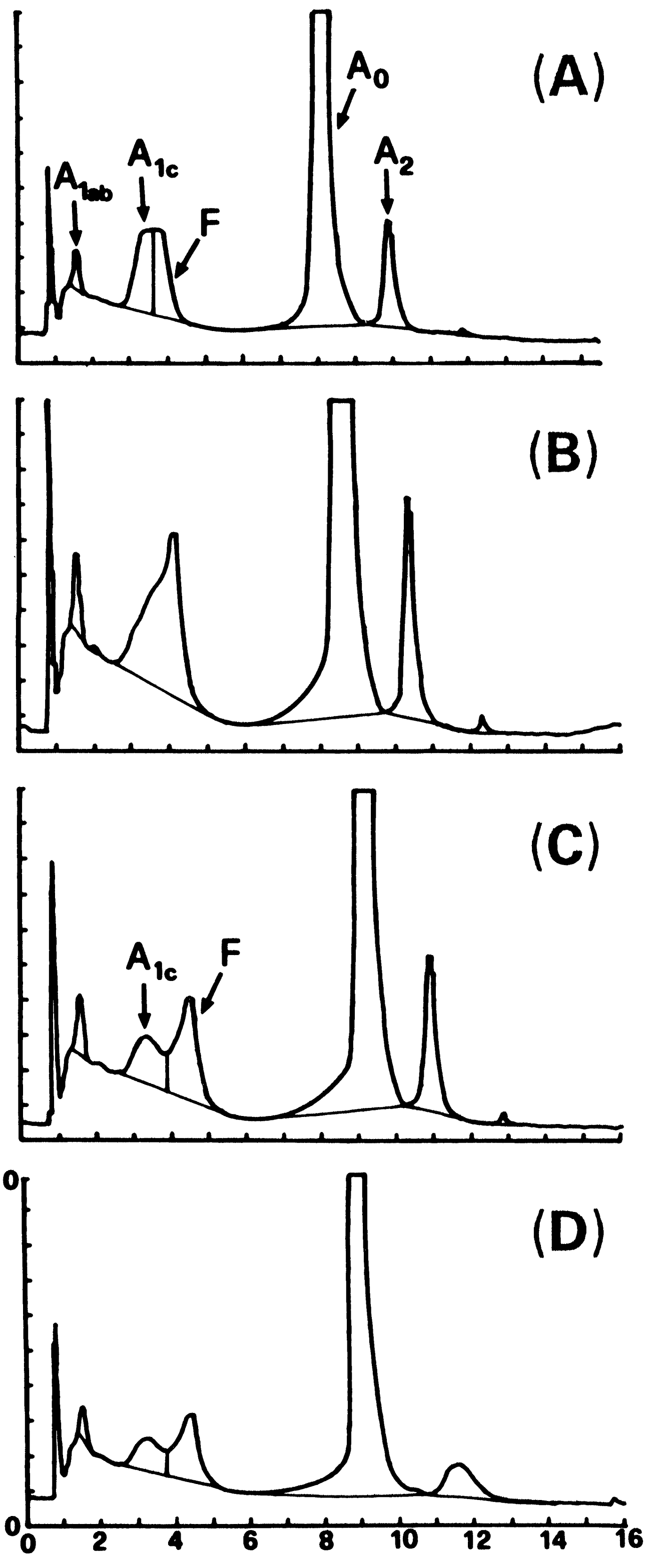

*Figure 1. Effect of the elongation of buffers elution time (e.t.) on the resolution between Hb $A_{1c}$ and Hb F. (a): instrumental parameters as recommended by Bio-Rad Laboratories; (b)–(d): tested modifications. (a): Buffer 1 e.t.: 1·0 min; buffer 2 e.t.: 6·4 min. (b): Buffer 1 e.t.: 1·5 min; buffer 2 e.t.: 6·4 min. (c): Buffer 1 e.t.: 2·0 min; buffer 2 e.t.: 6·4 min. (d): Buffer 1 e.t.: 2·0 min; buffer 2 e.t.: 9·4 min. The ordinate axis (absorbance) is automatically set to a fixed percentage of full scale (typically 10%), when Hb $A_{1c}$ is precisely located. The* x-*axis is the elution time (min). The scales of graphs (a)–(c) are the same as for (d).*

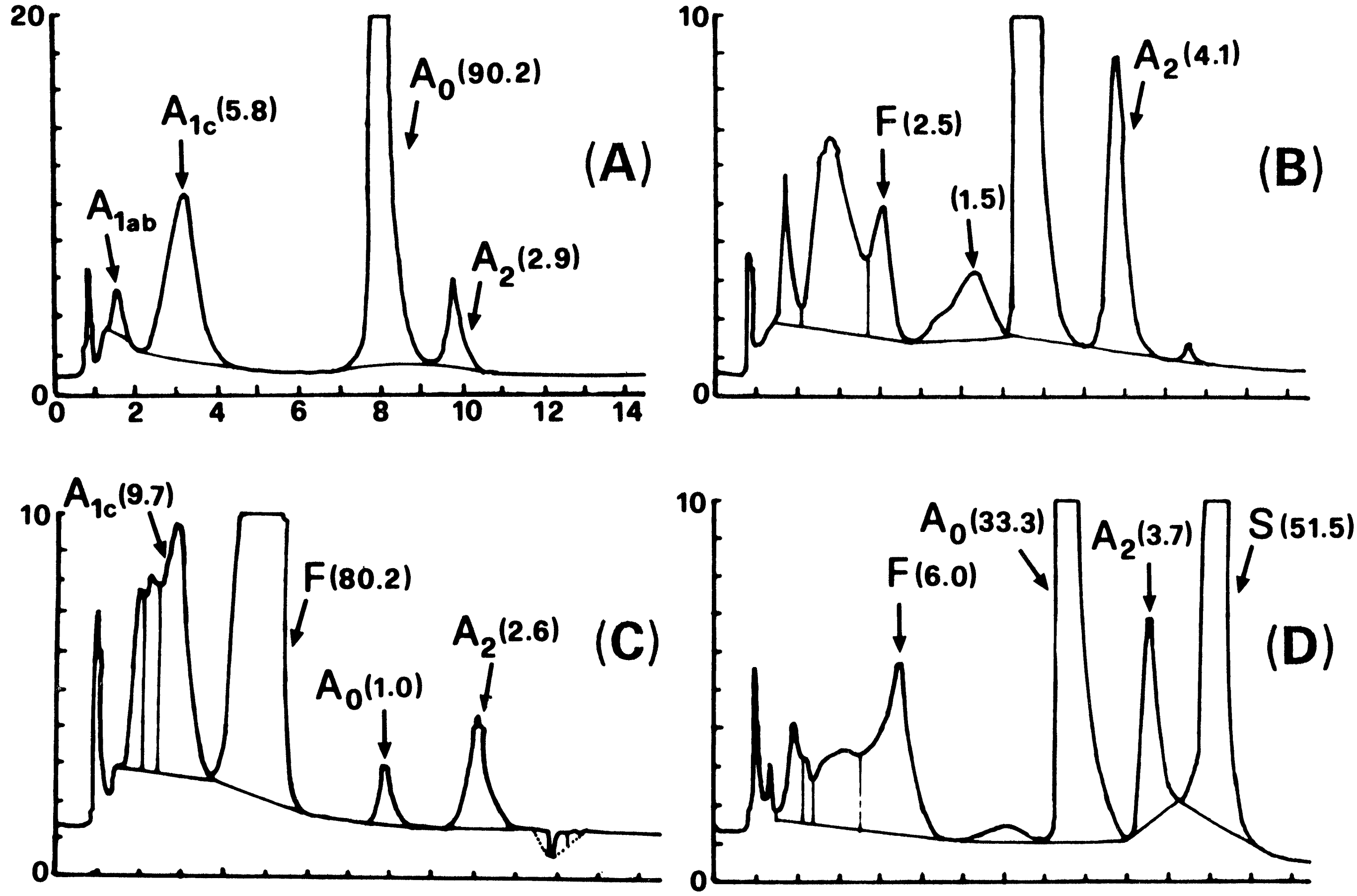

*Figure 2. Chromatograms obtained from the analysis of a normal subject (a), a β-thalassaemia carrier (b), a β°-homozygous (c) and a double heterozygous for β-thal and Hb S (d). The relative concentrations of the different haemoglobin components are reported in parentheses, in % unit. (b), (c) and (d)* x*-axis scales as in (a).*

As Hb $A_{1c}$ reference method another Diamat instrument was used, which was dedicated to Hb $A_{1c}$ measurement for the diabetic centre (later called the x-method). This instrument is equipped with a different column and buffer elution system; and its analytical characteristics have previously been evaluated [12–14]. Each blood sample was therefore treated independently for each analysis and loaded on the two instruments almost simultaneously (or at least no longer than 12 h between the two determinations).

*Statistical analysis*

All the statistics have been calculated on an IBM personal computer by using parametric tests. Hb $A_2$ values in normals and in β-thal carriers were found to be normally distributed, as evaluated by Kolmogorov-Smirnov test [18] and asymmetry and curtosis coefficients. The determination of the reference limits was performed according to the IFCC recommendations [19].

### Results

*Chromatographic resolution of minor and abnormal haemoglobins*

Some typical chromatograms obtained by the Diamat analyser are reported in figures 2 and 3. As can be seen from figure 2(*a*), Hb F is normally not resolved if its concentration is below 1%. When an abnormal peak is isolated, this is reported as an unknown fraction which has to be interpreted from the chromatogram. The small peak between Hb F and Hb $A_0$ (figures 2[*b*] and [*d*]) is usually absent, and, if present, never exceeds 1·5% of total haemoglobin. This can probably be identified as Hb $A_{1d}$, an adduct of haemoglobin and oxidized glutathione [20].

Surprisingly, in the case of β-thalassaemic homozygous (figure 2[*c*]) a small percentage of Hb $A_0$ and an abnormal Hb $A_{1c}$ peak was found. Complete absence of Hb $A_0$ in this subject has been demonstrated by DNA analysis with synthetic oligonucleotides [21], which showed a mutation at codon β39 (CAG → TAG). This kind of mutation is responsible for more than 95% of all cases of β-thalassaemia in Sardinia. Therefore, the small fraction detected as Hb $A_0$ is probably an artefact, or some unidentified component.

As regards to Hb $A_{1c}$, a significant amount of acetylated Hb F is probably responsible for the elevation of the peak; from a comparative study with isoelectric focusing on cord blood samples, it has been shown that Hb F is eluted among Hb $A_1$ fractions (data not shown here).

Hb H and Hb Bart's (figures 3[*a*] and [*b*]) are eluted before the Hb $A_1$ fractions; this is in agreement with data

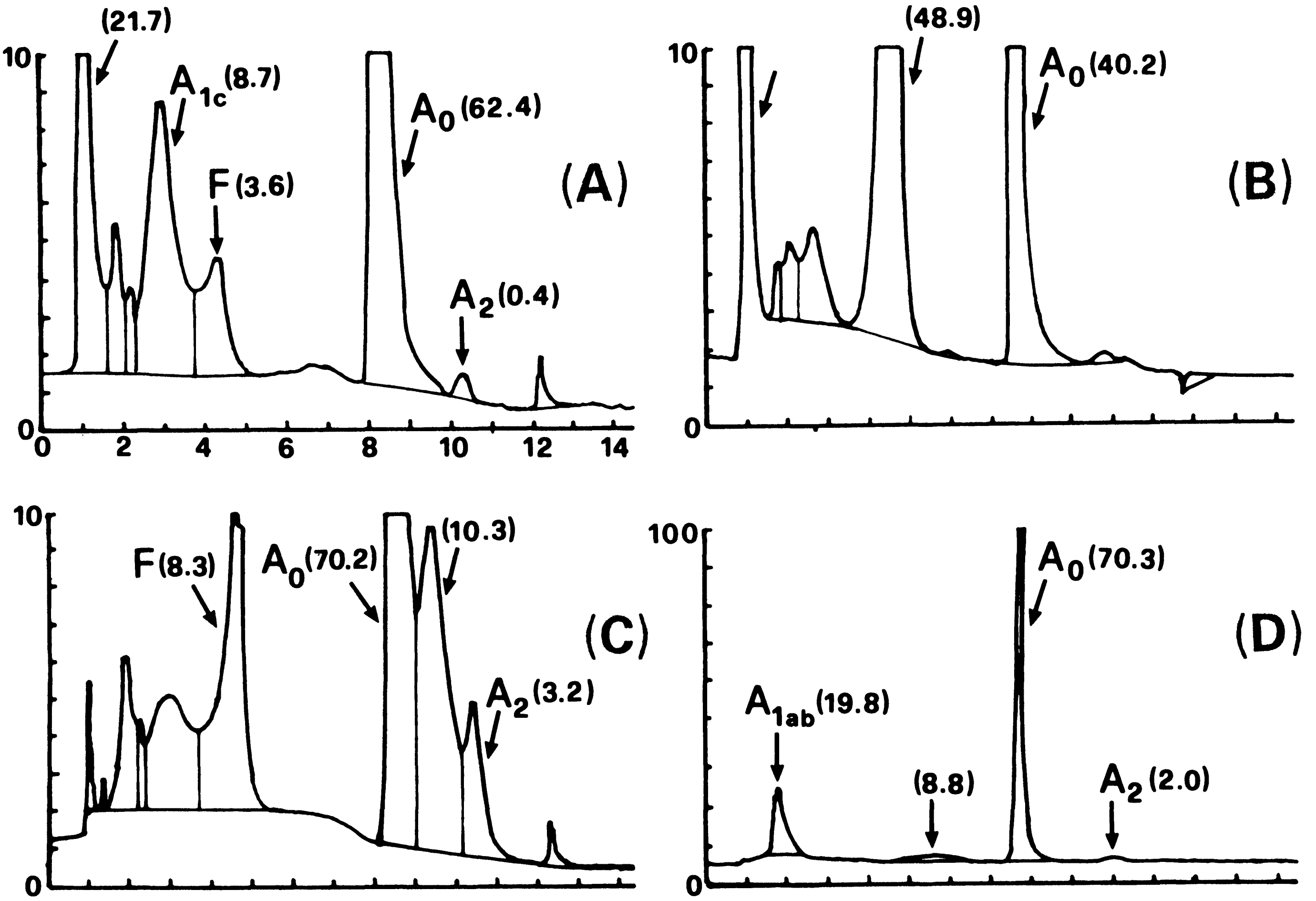

*Figure 3. Chromatograms from: a subject suffering from Hb H disease (a), a newborn with Hb Bart's (b), an Hb Lepore (c) and an Hb J Sardegna carrier (d).*

reported when using a similar cation-exchange HPLC procedure [20]. Hb Lepore (figure 3[*c*]) is slightly underestimated with respect to densitometric analysis (10·3% versus 15·0%, respectively).

Figure 3(*d*) reports on a case of a Hb J Sardegna (α50 [CE8] His → Asp) carrier; as frequently happens in such cases, if the Hb $A_{1c}$ is not resolved, the absorbance scale is automatically set to 100%. The haemoglobin variant is eluted among the Hb $A_I$ fractions in agreement with its 'fast' electrophoretic mobility and is correctly quantitated (about 19%, against the 20·5% obtained by densitometry of the electrophoretic trace).

Accuracy of Hb $A_{1c}$ determination has been tested by analysing several samples, from normal and diabetic subjects, using the two Diamat procedures. The results of such a comparison are reported in figure 4.

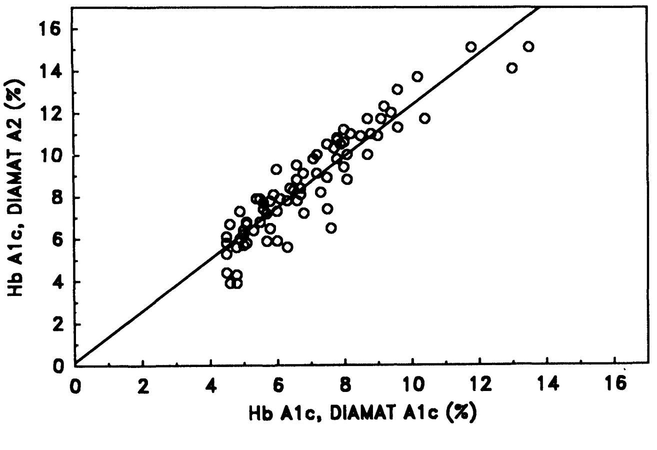

*Figure 4. Comparison between the present method (y) and a reference method (x) for Hb $A_{1c}$ quantification: $y = 1{\cdot}217x + 0{\cdot}16$; $n = 79$; $r = 0{\cdot}9235$; $S_{yx} = 0{\cdot}99$.*

*Analytical performance*

Total imprecision of the method was tested separately for Hb $A_2$ and Hb F by analysing several samples from normals and β-thal carriers. The results are reported in table 1. The Hb $A_2$ determination is highly reproducible (CV never exceeding 4%). Acceptable imprecision has also been found for Hb F; only for concentrations in the range of 1% or lower are the CVs significantly higher.

Linearity was evaluated by measuring in duplicate different haemoglobin solutions prepared by mixing Hb

*Table 1. Analytical imprecision.*

| | Hb $A_2$ | | | Hb F | | |
|---|---|---|---|---|---|---|
| | *N* | CV Mean ± SD | % | *N* | CV Mean ± SD | % |
| Within-Run | | | | | | |
| Normal | 10 | 2·71 ± 0.07 | 2·6 | 10 | 1·30 ± 0·07 | 5·1 |
| Abnormal-1 | 10 | 5·22 ± 0·15 | 2·9 | 10 | 3·47 ± 0·14 | 4·1 |
| Abnormal-2 | — | — | — | 10 | 7·94 ± 0·20 | 2·5 |
| Between-run | | | | | | |
| Normal | 15 | 2·82 ± 0·10 | 3·6 | 17 | 0·71 ± 0·19 | 27·0 |
| Abnormal | 15 | 4·03 ± 0·16 | 3·9 | 22 | 3·91 ± 0·13 | 3·2 |

$A_0$ and Hb $A_2$, isolated by conventional liquid chromatography (Hb $A_2$ linearity test) or saline washed erythrocytes obtained from a normal subject and cord blood (Hb F linearity test).

The HPLC procedure for these measurements is linear for Hb $A_2$ concentrations up to 6% ($y = 0·94x + 0·45$; $r = 0·992$; $S_{yx} = 0·22$) and for Hb F values up to 34% ($y = 1·42x - 0·37$; $r = 0·998$; $S_{yx} = 0·88$). However, acceptable Hb F measurement can be taken even at higher Hb F concentrations, up to 60% ($y = 1·08x + 2·44$; $r = 0·990$; $S_{yx} = 3·36$).

Accuracy was tested by making a comparison of the Diamat proposed procedure against several reference methods, as reported in table 2. The correlations between these methods were excellent.

Finally, the procedure has no carry-over and is insensitive to changes in sample total haemoglobin, in the range 4·4–20·4 g/dl (data not reported in detail).

With regards to column life, we found that Hb F can be properly resolved for up to 750–800 runs. Up to 1100 runs of Hb $A_2$ can be made.

*Reference values*

Hb $A_2$ reference values were evaluated for normal adults and β-thal carriers. From the statistical analysis of elementary data the following values were obtained:

| | | | |
|---|---|---|---|
| (1) | Normals ($N$ = 597) | Mean ± SD: | 2·64 ± 0·32 |
| | | 95% confidence interval: | 2·02 – 3·27% |
| (2) | β-thal carriers ($N$ = 200) | Mean ± SD: | 4·91 ± 0·51% |
| | | 95% confidence interval: | 3·92 – 5·90%. |

The cut-off limit can be set at Hb $A_2$ = 3·35%; out of the 797 subjects only three had Hb $A_2$ values between 3·4 and 3·6%.

Hb F values in normals and in different thalassaemia syndromes carriers were distributed as follows. Hb F concentrations higher than 1·0% were measured in:

(*a*) 44 out of 192 normal young subjects (age 1–14 years, 22·9%) and in 41 out of 776 normal adults (5·3%).

(*b*) 38 out of 65 β-thal young carriers (age 1–14 years, 58·5%) and in 81 out of 237 β-thal adults (34·2%).

Hb F concentrations in three non-deletion δβ-thalassaemia carriers, Sardinian type [22] were: 14·1 ± 1·7% (mean ± SD).

### Discussion

The authors first experiences with the Diamat system applied to the determination of haemoglobin species in blood are reported. It is clear that this apparatus can improve routine haematological management in the clinical laboratory. The system offers the following advantages:

(1) Hb $A_2$ is measured accurately and reproducibly. This is relevant because Hb $A_2$ accounts for only a small amount of total haemoglobin (Hb $A_2$ concentrations higher than 6% are rarely found) and because the separation limit between normals and β-thalassaemics is very narrow. A similar reproducibility is rarely obtained with manual chromatographic methods.

The reference intervals found for normals and β-thalassaemia carriers are in agreement with those already reported [3].

*Table 2. Relevant linear regression parameters concerning the comparison among the Diamat method (y) and different techniques for Hb $A_2$ and Hb F determination (x-methods).*

| | x-method | *N* | Slope ± SD | Int. ± SD | $S_{yx}$ | Range, % | *r* |
|---|---|---|---|---|---|---|---|
| Hb $A_2$ | Ion-exchange | 70 | 0·883 ± 0·030 | 0·33 ± 0·12 | 0·30 | 2·0–6·0[a] | 0·9639 |
| | Ion-exchange (Helena) | 200 | 0·942 ± 0·015 | 0·36 ± 0·06 | 0·28 | 1·6–5·9[a] | 0·9755 |
| Hb F | Alkali den.[c] | 30 | 1·078 ± 0·011 | −0·60 ± 0·20 | 0·98 | 1·3–55·0[b] | 0·9990 |
| | Alkali den.[d] | 49 | 1·016 ± 0·005 | −0·12 ± 0·23 | 0·83 | 12·0–80·0[b] | 0·9995 |
| | Radial imm.[d] | 31 | 1·040 ± 0·031 | 0·17 ± 0·17 | 0·52 | 0·5–12·0[b] | 0·9877 |
| | Diamat-$A_{1c}$ | 31 | 1·030 ± 0·013 | 0·20 ± 0·30 | 1·39 | 1·1–65·0[b] | 0·9980 |

a: Minimum and maximum Hb $A_2$ concentrations determined by x-method.
b: Minimum and maximum Hb F concentrations determined by x-method.
c: According to ref. [11].
d: According to ref. [9].

(2) Simultaneous Hb F determination eliminates the need for separate analysis; this kind of measurement is, for most Hb F assay protocols, time-consuming, poorly reproducible and seldom linear to high concentrations.

(3) Chromatographic analysis is able to discriminate between several haemoglobin variants; their quantifications is, in the majority of cases, in agreement with the electrophoretic pattern.

(4) Sampler capacity (48 tubes) and the system's automation allow up to 90 samples per day to be analysed; no special skills are required to operate the instrument.

A cation exchange HPLC analysis recently proposed by Bisse and Wieland [20] seems to offer similar advantages. The system optimized by these authors is probably superior, in terms of resolution, but compared to the Diamat procedure, has the following drawbacks:

(*a*) The analysis is more time-consuming (each run takes more than 60 min, while the Diamat run is only one-third as long).

(*b*) No information is available on accuracy in Hb $A_2$ measurement.

(*c*) Hb $A_{1c}$ accuracy has been evaluated on a small number of subjects ($N = 25$), with only three of these subjects out of normal range.

No other fast high resolution HPLC procedure for the separation of Hb F, Hb $A_0$, Hb $A_2$ and other Hb variants seems to have been presented.

The Diamat system has some minor problems. For example Hb $A_{1c}$ quantification seems to be overestimated with respect to a reference method of proved accuracy [12]. A possible explanation for this could be the influence of labile aldimine forms which, if not removed during the haemolysis step, can co-migrate with the Hb $A_{1c}$ component (stable ketoamine form). The importance of removing these labile forms has been stressed by several investigators [23–25]. It has also been demonstrated that the Diamat $A_{1c}$ analysis (the x-method in figure 4) is specific for the stable forms [25]. In fact, a significative difference has been found by analysing the haemolysing agent used for Hb $A_{1c}$ determination (x-method) and the one used with the test method (y-method in figure 4); the two agents differ in pH (5·95 versus 7·20, respectively) and in conductivity (246 versus 132 μS, respectively). In order to confirm that the haemolysing agent has a powerful effect on Hb $A_{1c}$ concentration, Hb $A_{1c}$ was measured using the proposed method, in a sample treated with the two haemolysing agents separately. The following values were found: 5·3% (x-method haemolysing solution) and 7·3% (y-method haemolysing solution). In conclusion, the Hb $A_{1c}$ measurement on Diamat equipped with reagents and column for Hb $A_2$ and Hb F is probably inaccurate, because the labile aldimine form is not removed during the haemolysis step.

A second limitation of the technique concerns peak identification. This is easily done for normal samples, but, when dealing with abnormal samples (for example those reported in figures 2 and 3) peak identification should be performed by an experienced haematologist. It is also evident that, if a pathological result is obtained, further analyses, such as DNA analysis and globin chain synthesis, have to be made in order to obtain a final diagnosis.

Apart from these limitations, this kind of HPLC analysis is useful for diagnosis of haemoglobin-related disorders. Improvements, such as a further reduction of analysis times for a correct estimation of Hb $A_{1c}$, would be useful.

### Acknowledgements

The authors gratefully acknowledge Professor A. Cao (Universita' degli Studi, Cagliari, Italy) for helpful discussions and suggestions and Dr L. Benazzi (CNR, Milan, Italy) for his critical evaluation of the manuscript.

### References

1. Weatherall, D. J., and Clegg, J. B., *The Thalassaemia Syndromes*, 3rd edition (Blackwell Scientific, Oxford, 1981), 744.
2. Bertles, J. F., *Annals of the New York Academy of Sciences*, **241** (1974), 638.
3. International Committee for Standardization in Haematology (ICSH), *British Journal of Haematology*, **38** (1978), 573.
4. Ou, C. N., Buffone, G. J., Reimer, G. L., and Alpert, A. J., *Journal of Chromatography*, **266** (1983), 197.
5. Gupta, S. P., and Hanash, S. M., *Analytical Biochemistry*, **134** (1983), 117.
6. Turpeinen, U., *Clinical Chemistry*, **32** (1986), 999.
7. Bardakdijan, J., Vallez, J. M., Rosa, J., and Galacteros, F., Second International Conference on Thalassemia and the Hemoglobinopathies held in Crete, Greece (1987), p. 60.
8. Chudwin, D. S. and Rucknagel, D. L., *Clinica Chimica Acta*, **50** (1974), 413.
9. Singer, R., Chernoff, A. I., and Singer, L., *Blood*, **6** (1951), 413.
10. Betcke, K., Marti, H. Q., and Schlicht, I., *Nature*, **184** (1959), 1877.
11. Molden, D. P., Alexander, N. M., and Neeley, W. E., *American Journal of Pathology*, **77** (1982), 568.
12. Carpinelli, A., Mosca, A., and Bonini, P. A., *Journal of Automatic Chemistry*, **8** (1986), 192.
13. Brenna, S., Prencipe, L., Granata, S., and Montalbetti, N., *Journal of Clinical Chemistry and Clinical Biochemsitry*, **25** (1987), 437.
14. Davids, J. A., Goodman, J., and Naito, H. K., *Clinical Chemistry*, **34** (1988), 1301.
15. Bruegger, B., Keenan, D. H., and Sivorinovsky, G., *Clinical Biochemistry*, **21** (1988), 363.
16. Paleari, R., Arcelloni, C., Paroni, R., Fermo, I., and Mosca, A., *Clinical Chemistry*, **35** (1989), 425.
17. Galanello, R., Melis, M. A., Muroni, P., and Cao, A., *Acta Haematologica*, **57** (1977), 32.
18. Snedecor, G. W., and Cochran, W. G., *Statistical Methods*, 7th edition (Iowa State University Press, Ames, Iowa, 1980), 492.
19. Solberg, H. E., *Journal of Clinical Chemistry and Clinical Biochemistry*, **21** (1983), 749.

20. Bisse, E., and Wieland, H., *Journal of Chromatography,* **434** (1988), 95.

21. Rosatelli, C., Falchi, A. M., Tuveri, T., Scalas, M. T., di Tucci, A., Monni, G. and Cao, A., *Lancet,* i (1985), 241.

22. Pirastu, M., Kan, Y. W., Galanello, R. and Cao, A., *Science,* **223** (1984), 929.

23. Compagnucci, P., Cartechini, M. G., Bolli, G., de Feo, P., Santeusanio, F. and Brunetti, P., *Diabetes,* **30** (1981), 607.

24. Nathan, D. M., *Clinical Chemistry,* **27** (1981), 1261.

25. Carenini, A., Mosca, A. and Pozza, G., *Clinical Chemistry,* **29** (1983), 1687.

### NOTES FOR AUTHORS

*Journal of Automatic Chemistry* covers all aspects of automation and mechanization in analytical, clinical and industrial environments. The *Journal* publishes original research papers; short communications on innovations, techniques and instrumentation, or current research in progress; reports on recent commercial developments; and meeting reports, book reviews and information on forthcoming events. All research papers are refereed.

### Manuscripts

Two copies of articles should be submitted. All articles should be typed in double spacing with ample margins, on one side of the paper only. The following items should be sent: (1) a title-page including a brief and informative title, avoiding the word 'new' and its synonyms; a full list of authors with their affiliations and full addresses; 12) an abstract of about 250 words; (3) the main text; (4) appendices (if any); (5) references; (6) tables, each table on a separate sheet and accompanied by a caption; (7) illustrations (diagrams, drawings and photographs) numbered in a single sequence from 1 upwards and with the author's name on the back of every illustration; captions to illustrations should be typed on a separate sheet. Papers are accepted for publication on condition that they have been submitted only to this *Journal.*

### References

References should be indicated in the text by numbers following the author's name, i.e. Skeggs [6]. In the reference section they should be arranged thus:

to a journal

Manks, D. P., *Journal of Automatic Chemistry,* **3** (1981), 119.

to a book

Malmstadt, H. V., in *Topics in Automatic Chemistry,* Ed. Stockwell, P. B. and Foreman, J. K. (Horwood, Chichester, 1978), p. 68.

### Illustrations

Original copies of diagrams and drawings should be supplied, and should be drawn to be suitable for reduction to the page or column width of the *Journal,* i.e. to 85 mm or 179 mm, with special attention to lettering size. Photographs may be sent as glossy prints or as negatives.

### Proofs and offprints

The principal or corresponding author will be sent proofs for checking and will receive 50 offprints free of charge. Additional offprints may be ordered on a form which accompanies the proofs.

*Manuscripts should be sent to Dr P. B. Stockwell, P.S. Analytical Ltd, Arthur House, Unit B4, Chaucer Business Park, Watery Lane, Kemsing, Sevenoaks, Kent TN15 6QY, UK.*

